# Classifications of Odontoid Process Fractures: A Systematic Review and Proposal of a New Simplified Classification System Based on Embryology

**DOI:** 10.7759/cureus.32520

**Published:** 2022-12-14

**Authors:** Mansour Mathkour, Juan J Cardona, Arada Chaiyamoon, Ryan M Glynn, Skyler Jenkins, Rachel A Graham, Jaspreet Johal, Brady Gardner, Joe Iwanaga, Aaron S Dumont, R. Shane Tubbs

**Affiliations:** 1 Neurosurgery, Tulane University School of Medicine, New Orleans, USA; 2 Neurosurgery, Ochsner Neuroscience Institute, Ochsner Health System, New Orleans, USA; 3 Neurosurgery Division, Jazan University, Riyadh, SAU; 4 Anatomy, Faculty of Medicine, Khon Kaen University, Khon Kaen, THA; 5 Orthopaedics, St. George's University, St. George's, GRD; 6 Internal Medicine, Sophie Davis School of Biomedical Education, New York, USA; 7 Neurology, Lehigh Valley Health Network, Philadelphia, USA; 8 Anatomical Sciences, St. George’s University School of Medicine, St. George’s, GRD; 9 Structural & Cellular Biology, Tulane University School of Medicine, New Orleans, USA; 10 Neurology, Tulane University School of Medicine, New Orleans, USA

**Keywords:** classification system, fractures, dens, odontoid process, axis, c2

## Abstract

Odontoid fractures are the most common cervical spine fractures in the elderly. Although many classification systems have been developed for them, the ambiguity in various definitions can potentially lead to misunderstandings. This paper aims to review the terminologies and current classification systems of odontoid fractures and propose a new, simplified anatomical classification. Given the descriptive variability of odontoid fractures in current classifications, we systematically reviewed the literature using PRISMA guidelines querying the National Library of Medicine PubMed database. The initial literature search yielded 175 publications. A total of seven reports met the inclusion criteria and were ultimately included for a full review. The classification systems previously used to categorize fractures of the odontoid process often need to be more transparent, imprecise, and incongruous. To simplify them, a new embryologically accurate system is proposed. A new embryological and anatomically-based system, combining the former systems' specific attributes, allows a more straightforward and adaptable classification of odontoid fractures.

## Introduction and background

Odontoid fractures account for 10-15% of all cervical vertebral fractures [1,2]. In patients over 70 years, fractures of the odontoid process are the most common cervical spine fractures [3]. The classification systems have been divided into categories according to fracture location, fracture direction, and odontoid process anatomy [4]. Each current classification system is located within that framework, yet some ambiguities and obscurities can potentially lead to misunderstandings.

The purpose of this paper is to review the current classification systems of odontoid fractures with their complexities, ambiguities, and drawbacks and to propose a new simplified anatomical classification based on the embryology of the C2 vertebra.

## Review

Methods

**Figure 1:**
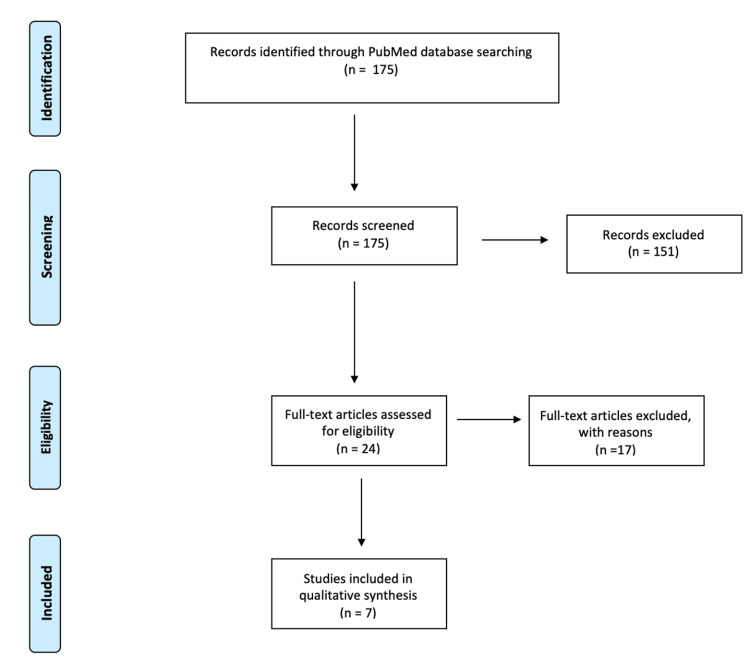
PRISMA flow chart of literature review PRISMA: Preferred Reporting Items for Systematic Reviews and Meta-Analyses.

We reviewed the literature systematically without meta-analysis by Preferred Reporting Items for Systematic Reviews and Meta-Analysis (PRISMA) statement [5] regarding current classifications of odontoid fractures, querying the National Library of Medicine PubMed database using the combination of MeSH terms "Odontoid fracture OR Dens fracture" AND "Classification." Abstracts were then screened using the inclusion criteria of English reports discussing odontoid fracture classification. The search period ranged from 1970 to 2022. We excluded articles that (1) did not mention fractures relating to the odontoid process or dens or (2) focused solely on surgical treatment strategies and outcomes (Figure 1).

Results

The initial literature search yielded 175 publications. Of these, 24 were found to meet the inclusion criteria and were subjected to review for exclusion. From the past 52 years, seven reports were ultimately selected for full review since 17 were found irrelevant owing to one or both points listed in the exclusion criteria. A search flow diagram is provided in Figure 1. All of the included reports discussed unique proposed classification systems for odontoid fractures.

Korres et al. [4] divided the classification systems into three separate categories depending on if their classification was based on: (1) fracture location and position, (2) fracture direction, or (3) odontoid process anatomy. Each of the following systems is located within that framework. The Schatzker [6], Althoff [7], and Anderson-D'Alonzo systems [8] fall under the umbrella of fracture position. Grauer et al. [9] and Hadley et al. [10]independently subclassified the Anderson-D'Alonzo system. The Roy-Camille system classified odontoid fractures based on fracture direction [11].

This report discusses the previous classification systems in detail with their advantages and drawbacks (Table 1). Additionally, we propose a new simplified classification system based on the embryological development of the odontoid process and its anatomy and guide clinical decision-making in their management.

**Table 1:** Summary of current and previous odontoid fracture classification systems with their drawbacks

System	Based on	Classification	Drawback
Schatzker’s (1971) et al. [6]	Fracture position and location in relation to the attachment of the accessory ligaments	Low fracture either at the level or below the level of the attachment of the accessory ligaments	1. Diversity of terminologies surrounding the location of the fracture 2. Some lack of internal fracture morphology and type of fracture 3. Discrepancies regarding fracture stability, comminution and displacement which ultimately affect management recommendation 4. Often no distinct anatomic borders defined between fracture types
		High fracture on one or both sides, above the attachment of the accessory ligaments	
Anderson and D’Alonzo’s (1974) et al. [8]	Fracture position and location	Type I oblique fracture at the tip of the odontoid process	
		Type II at the base of the dens and the junction with the body	
		Type III at the junction of the dens with the anterior portion of the axial body extending into the body and lateral masses of C2	
Althoff’s (1979) et al. [7]	Fracture position and location	Type A above the neck	
		Type B through the neck including superior C2 body	
		Type C includes the medial part of one C2 superior articulating process	
		Type D includes fractures through the body of C2, including the medial part of both superior articulating processes	
Hadley’s (1988) et al. [10]	Subclassification of Anderson and D’Alonzo’s	Type I and type III the same as Anderson and D’Alonzo’s	
		Type IIA additional bone fragment at the dens fracture site	
Grauer’s (2005) et al. [9]	Subclassification of Anderson and D’Alonzo’s	Type I and type III same as Anderson and D’Alonzo’s	
		Type IIA not displaced from their site of origin	
		Type IIB displaced and/or extend transversely from anterior superior to posterior inferior	
		Type IIC comminuted and/or extend from anterior inferior to posterior superior	
Roy-Camille’s (1979) et al. [11]	The direction of the fracture line and the amount of displacement	Type I obliquely slanted from posterior to anterior displacing the odontoid process anteriorly	1. Fails to describe fracture location 2. Did not address the relationship of fractures involving the body of C2
		Type II oblique angle slants anterior to posterior displacing the odontoid process posteriorly	
		Type II horizontal fracture with no angulation displacing the odontoid process anteriorly or posteriorly	
Current system	Embryological development and anatomy of the odontoid process	Type I involving the upper ¼ of the dens (zone I)	1. Only fractures involving and related to the anatomical odontoid process
		IA non displaced	
		IB displaced and/or angulated odontoid process anteriorly or posteriorly	
		Type II involving the lower ¾ of the dens (zone II)	
		IIA non displaced	
		IIB displaced and/or angulated odontoid process anteriorly or posteriorly	

Discussion

Embryology and Odontoid Development

**Figure 2:**
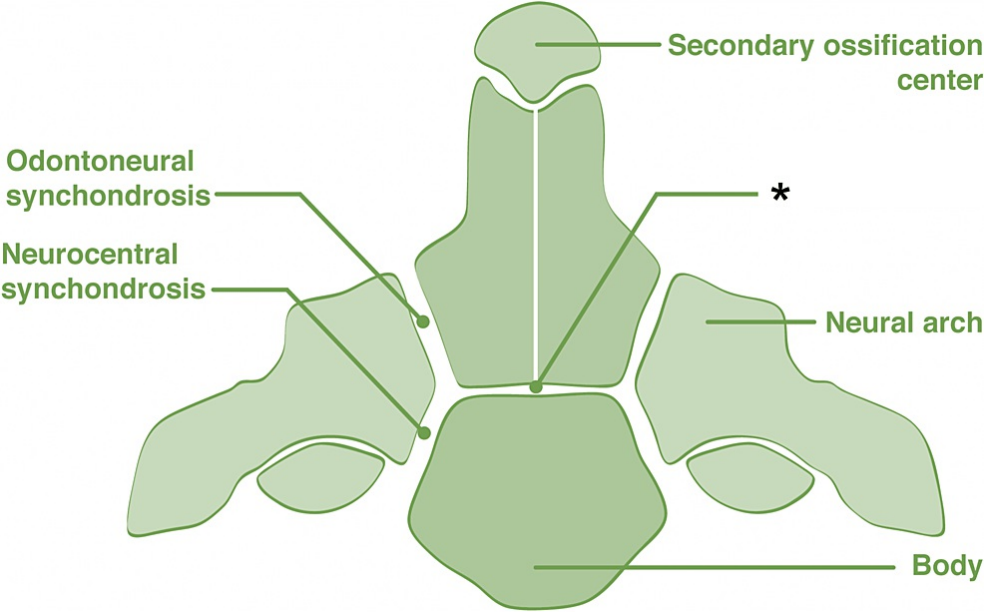
Coronal view of the C2 vertebra noting its embryological fusion sites. The subdental synchondrosis is seen at the * Author's own work.

**Figure 3:**
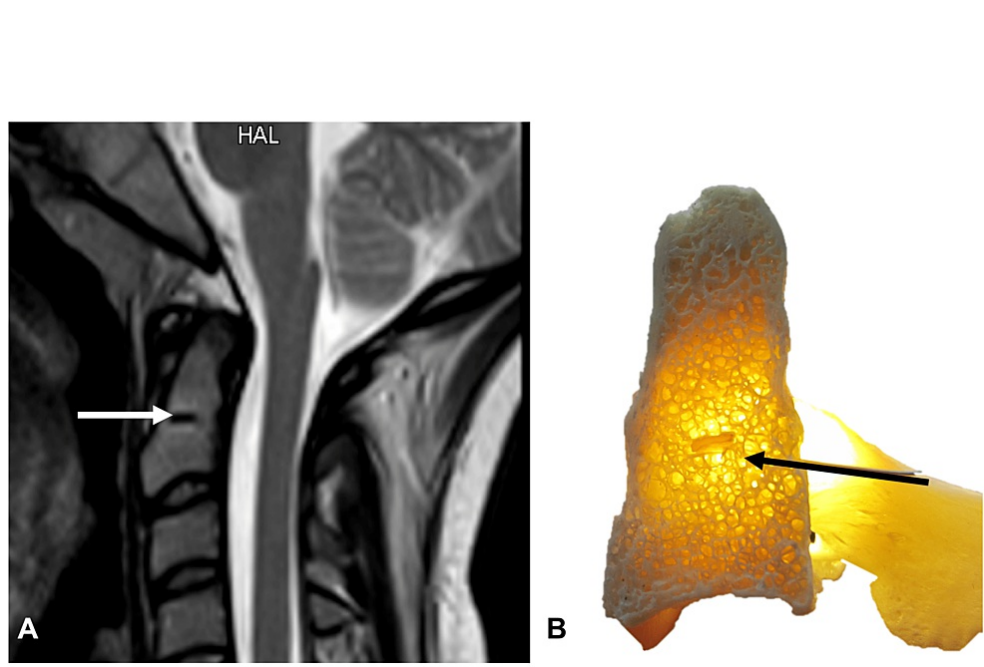
A: MRI and B: dry bone specimen noting the subdental synchondrosis (arrows). Author's own work.

The odontoid process of C2 (dens) has been widely studied because of its anatomical importance. Formerly thought to originate as a displaced body of C1 (atlas), it is now accepted that the dens separate from the anterior arch of C1, moving inferiorly to fuse with the axis during the sixth and seventh weeks of gestation [12]. The dens are derived from two separate ossification centers. The primary ossification centers form laterally at the base of the odontoid, which comprises the union between the two primary ossification centers and fuses in the midline by the eighth month of fetal life. The secondary ossification center (called the cuneiform cartilage) forms the apex/tip of the odontoid process (Figure 2). The subdental synchondrosis develops between the primary ossification centers of the odontoid process and the corpus of C2 (Figure 3), which remains visible on radiographic imaging until the 11th year of life [12,13]. There is significant variability in research concerning the timeline for the persistence of these synchondroses through adulthood, as Jenkins et al. [14] found in as few as 25% of cases. In contrast, Gebauer et al. [13] detected them in almost 90%. The development and morphology of the odontoid process provide a foundation for classifying odontoid fractures and their treatment options.

Odontoid Process Anatomy and Nomenclature

The odontoid process is a conical projection from the C2 vertebral body and serves as an attachment site for the cranioatlantoaxial ligamentous complex [12,15]. It is often referred to as the dens, odontoid peg, or processus epitrophysis [12,16,17]. The tip of the odontoid process extends from the apex of the dens to the apicodental junction.

The apex gives origin to the apical ligament, which extends rostrally to attach to the basion. The atlas' anterior arch articulates with the odontoid process's anterior surface [15]. The broad posterior groove and the posterolateral surfaces are attachment sites for the transverse and alar ligaments [12,16,17].

**Figure 4:**
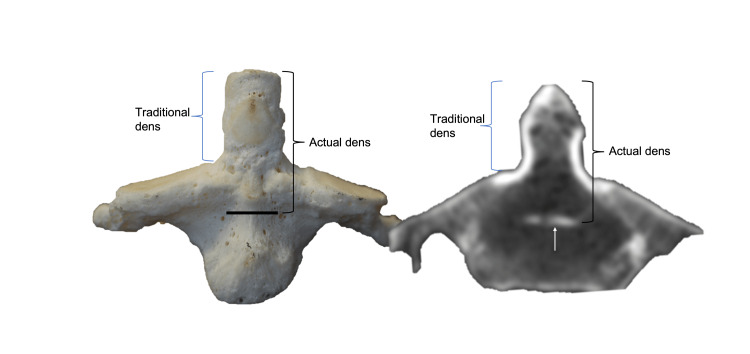
Anterior view of C2 vertebra on skeletal specimen and CT noting the subdental synchondrosis and the traditional and actual extents of the dens. Author's own work.

**Figure 5:**
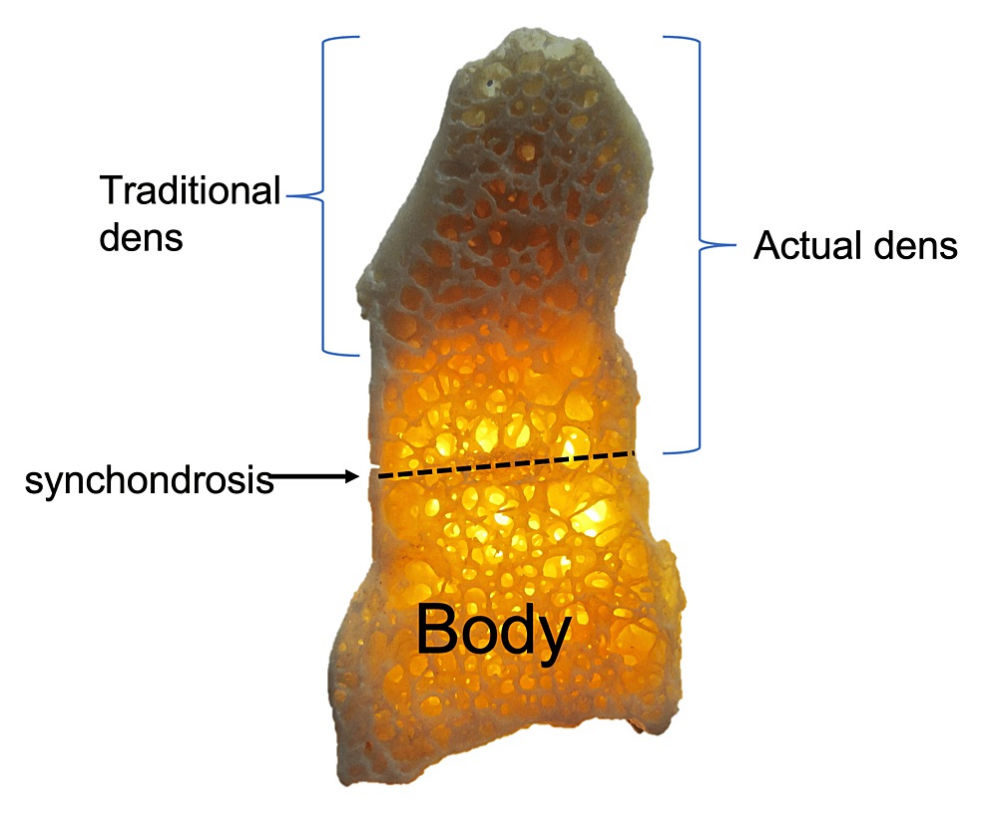
Sagittal view of C2 vertebra on skeletal specimen noting the subdental synchondrosis and the traditional and actual extents of the dens. Author's own work.

The bone between the apicodental junction and a horizontal line connecting bilateral superior articular processes forming the neck of the dens is known as the chondrum terminale [18,19]. The base or waist of the dens is where it attaches to the C2 vertebral body with the narrowest transverse diameter, just superior to the body of C2 (Figures 4, 5) [8,20-22].

However, others have defined the base as the bone between the part of the dens found along a horizontal line connecting bilateral superior articular processes and the subdental synchondrosis, also known as the dentocentral junction or odontocentral synchondrosis (Figures 4, 5) [18]. The terms waist and neck are used interchangeably for the part where the transverse ligament passes posteriorly [23]. The connection between the waist and tip of the dens has also been described as the neck, the stem, or the shaft of the dens, which is connected inferiorly to the C2 vertebra centrum at the subdental synchondrosis [24,25].

Schatzker's Classification System

In 1971, Schatzker's classification divided the dens into two types according to where the accessory ligaments are attached. The fractures could be above or below those ligaments; a low fracture is either at the level of the attachment or below it, while a higher fracture involving either one or both sides is above the attachment [6].

Anderson and D'Alonzo's Classification System

**Figure 6:**
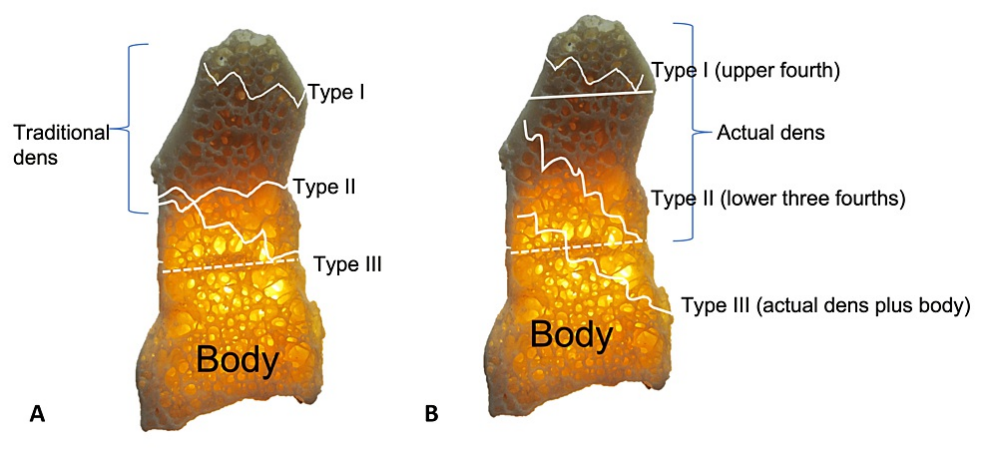
A: Sagittal view of C2 vertebra noting fractures and using a traditional system to describe these injuries. B. Application of the new simplified system (Tulane classification) of describing odontoid fractures. The upper horizontal line (right) approximates the apicodental synchondrosis, and the lower dotted lines represent the subdental synchondroses. Author's own work.

D'Alonzo and Anderson [8] in 1974 proposed that odontoid fractures can be categorized into Types I, II, or III according to their anatomic location (Figure 6A). Type I fractures, which are the least common, occur at the tip of the odontoid process [8]. Type II fractures are the most common, accounting for 65-74% of cases, and occur at the base of the dens causing subluxation of the atlas on C2 [3,8]. The etiology of these fractures is age-related; as younger patients present due to high loading, for instance, in motor vehicle accidents; whereas in patients aged over 60, these fractures tend to result from hyperflexion/extension of the neck during low energy activities such as falling from an upright position [26]. This distinction is bolstered by studies showing a strong link between osteoporosis and odontoid fractures following minor trauma in the elderly [26,27]. Amling et al. [28] revealed that normal physiological degeneration of the dens entailed a loss of cortical thickness, decreased trabecular volume, and weakening of trabecular bone connectivity. Jenkins et al. [14] further developed this concept using microCT imaging to show that the trabecula was most dense between the subdental synchondrosis and the apex, the region of Type II fractures. Finally, type III fractures occur at the junction of the dens with the anterior portion of the axial body, extending into the lateral masses of the axis [8,14]. Generally, types I and III fractures are more stable, causing limited nerve involvement, and are often treated with non-invasive measures such as external mobilization [29,30]. Although this is the most commonly-used system, Barker et al. [31] found only "fair" interrater reliability and rejected their hypothesis that this system was reliable and reproducible.

Althoff’s classification system

In 1979, Althoff [7] proposed four types of fractures depending on the anatomic level. Type A is a fracture above the neck, Type B is through the neck, including the most superior aspect of the body of C2, Type C includes the medial part of one superior articular facet, and Type D includes fractures through the body of C2 including the medial part of bilateral superior articular facets. Types A-C have pseudoarthroses of 64%, 55%, and 50%, respectively, whereas Type D has a 97% rate of a union. In his initial study, the bony union was significantly increased with fractures involving the body of the axis and those displaced anteriorly compared to posterior displacement.

Roy-Camille's Classification System

Roy-Camille et al. [11,32] created a system based on the fracture line's direction and the displacement amount. Odontoid instability, and therefore patient prognosis, is related to the comminution and directionality of the fracture line. The authors classify a Type I fracture as obliquely slanted from posterior to anterior, allowing the dens to be displaced anteriorly. Similar to Type I, a Type II fracture's oblique angle slants anterior to posterior with possible posterior displacement of the dens. Type III is a horizontal fracture with no angulation, in which the dens can displace anteriorly or posteriorly. Finally, Type IV is described as a "policeman's hat fracture," a horizontal fracture extending through the C2 body, becoming transarticular. Predictive validity testing showed that this classification correlates with the speed of fracture healing, otherwise known as the rate of union [29].

Hadley's Classification System

Hadley et al. [10] also developed a subclassification of Anderson and D'Alonzo's classification system, specifically within the Type II fracture. This new description encompassed fractures with bone chips at the base of the dens fracture, which hinders proper re-alignment and bony union [10,33]. This subclass, accounting for <10% of odontoid fractures, led to non-union regardless of the initial amount of dens dislocation [10]. Because these often appear like Type II fractures, they were classified as Type IIA [10,33].

Grauer's Classification System

In 2005, Grauer et al. [9] proposed a modified classification system with descriptions very similar to Anderson and D'Alonzo's types I and III but with subclassification of type II fractures. Type IIA fractures were without displaced from their site of origin. Type IIB included fractures with oblique fractures from anterior superior to posterior inferior or with transverse displacement. Type IIC included fractures that extend from anterior inferior to posterior superior or were comminuted, but these do not include the superior articular facets. The advantage of this subclassification of Type II fractures was to guide treatment recommendations where type IIA underwent non-operative external immobilization, type IIB was considered for odontoid screw, and type IIC for posterior C1-2 fusion.

Drawbacks of Previous Classification System

The Anderson and D'Alonzo system can confuse owing to the diversity of terminologies surrounding the location of the fracture. A type I fracture can be depicted as an "apical (tip) fracture of the dens" [29], "an oblique fracture through the upper part of the odontoid" [30], or "located in the upper part of the odontoid process" [27]. This is further confused by Hadley's and Grauer's subclassification of Anderson-D'Alonzo class II; IIA, IIB, IIC. Because of the variety of descriptors, this classification can be confusing. In addition, their fracture types are solely anatomically based without further stratification of the fracture type, internal morphology, or degree of displacement.

The Roy-Camille system denotes the direction of the fracture and the displacement of the odontoid and predicts the rate of a union during treatment but fails to describe the location of the fracture. Also, it needs to precisely address the relationship between type 2 fractures and the body of C2. In this classification, some studies depict type II fractures as being at "the base of the odontoid process" [27]. In contrast, others describe them as "at the junction between the body of the odontoid process and the body of the C2 vertebra" [3]. Further clarity and consistency are needed about what is considered the base. To ensure reliability, the terminology used to depict these types should be unanimously agreed upon across each system.

Treatment options for odontoid fractures, including spinal fusion, depending on whether the fracture is stable or unstable. For instance, a Hadley type IIA fracture is considered unstable owing to the comminution, so treatment requires surgery to ensure union [33]. However, a Grauer type IIA fracture is characterized as non-displaced, so treatment is non-invasive, for example, by external immobilization [9]. The coalescence of nomenclature in classification systems can lead to confusion and could be more accurate, depending on the system under which the patient is placed.

Lastly, there are examples in the literature where none of the previous classifications apply. For instance, Adam et al. [30] described a patient with an odontoid fracture fitting none of those above criteria. The patient was a 91-year-old female who had suffered trauma from a low-energy fall. The fracture extended through the base of the odontoid and into the vertebral body, causing a 4 mm anterior-inferior displacement. The authors concluded that a new classification system should be created.

A New Simplified Classification System

**Figure 7:**
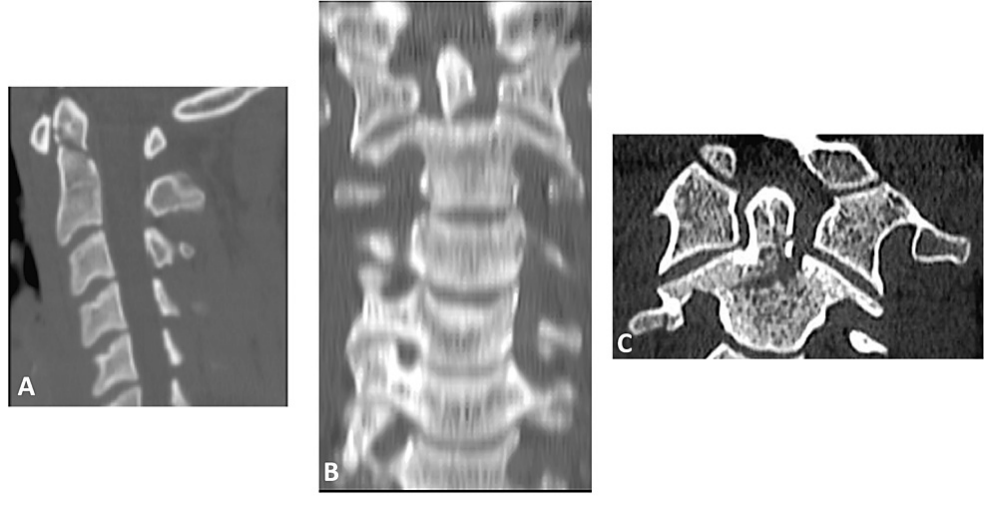
Examples of odontoid fractures. Using the new simplified system, A: Type I fracture. B: Type II fracture. C: Type III fracture.

A simplified and easily reproducible classification system originated from nomenclature proposed based on the developmental anatomy of the odontoid, developed by Johal et al. [34]. The apex of the dens develops from a unique secondary ossification center, while the remainder is formed from two laterally located primary ossification centers. The former does not necessarily fuse with the rest of the dens until puberty, and the latter forms the subdental synchondrosis. This classification system defines the dens anatomically from the superior part of the apex to the site of fusion with the body of C2 (at the synchondrosis). It consists of two zones. Zone I extends from the apex of the odontoid to the junction of the primary and secondary ossification centers (e.g., the upper ¼ of the dens or, i.e., the apicodental junction). Zone II extends from that junction to the synchondrosis (e.g., the lower ¾) [34]. This system allows for easy communication about the location of a fracture, on which further descriptors can be superimposed (Figures 6B, 7). For example, a fracture can be described as a "zone two oblique fracture with anterior displacement." Every party would then know that the fracture is located in the inferior ¾ of the dens.

Subclassification and Surgical Implications

Furthermore, the proposed classification can be subdivided according to whether the dens are displaced or angulated. If neither, we classify the fracture as type A; if there is any dens displacement and/or angulation either anteriorly or posteriorly, we can classify it as type B (Table 1). As techniques for odontoid fixation have evolved, management with either external or internal fixation depends on multiple factors, including fracture morphology, displacement, angulation, timing and stability of the fracture, integrity of the transverse ligament, patient age, and comorbidities, patient preference.

Therefore, both proposed types and subtypes can be considered unstable fractures, and we advocate surgical management for both. For type IA and type IIA and in the absence of contraindications, an anterior approach with an odontoid screw could be considered. Certain superior-type IA fractures may be managed with rigid external fixation at the surgeon's discretion. Contraindications for odontoid screw placement in type IA or IIA fractures most notably include transverse ligament disruption or type IIA with oblique type fracture. On the other hand, in type IB and type IIB, the surgical approach depends on the degree of angulation and displacement. In mild angulation and displacement cases, the anterior approach with an odontoid screw could be considered if there are no contraindications. These contraindications traditionally include anterior-inferior to the superior-posterior fracture line, transverse ligament injury, chronic fractures, pathologic fractures, comminuted internal morphology, or neck or chest anatomic limitations, including enlarged thoracic cavity diameter. In moderate to severe cases or in the presence of the above contraindications, the posterior approach with atlantoaxial fusion could be considered. The currently proposed system represents an evolution of the previous systems, considering the embryological development of the odontoid process and its anatomy. The proposal is only the first step for better communication and simplified classification. This system's prospective application to the classification and management of odontoid fractures will be required to evaluate and validate its utility.

Limitations

This proposed system has limitations. It would only include fractures related to the anatomical odontoid. Fractures involving the body of C2 would be excluded. This would remove any related confusion. Although the system does not address all the intricacies of related fractures and their underlying etiologies, it provides a reliable guide to the location of the fracture, which can be specified further [35-37]. Lastly, although not explored in the literature with comparative studies, ethnicity and sex could also play a role in the prevalence and type of fractures in this bone.

## Conclusions

Many classification systems for odontoid fractures have been developed, yet some ambiguities can potentially lead to misunderstandings. To help alleviate this ambiguity, we propose a simplified classification system (Tulane classification) that uses the embryological lines of development of the C2 vertebra as a foundation that can be built upon to describe odontoid fractures accurately. Prospective application of this system to both the classification and treatment of odontoid fractures will be required to validate its utility.
